# Yoga as a cost-effective adjunct therapy for dry eye: clinical outcomes from a 12-week randomized controlled trial

**DOI:** 10.3389/fmed.2026.1795593

**Published:** 2026-04-07

**Authors:** Ligi Abraham, Raghavendra Bhat, Sony George

**Affiliations:** 1 Division of Yoga and Life Sciences, Swami Vivekananda Yoga Anusandhana Samsthana (SVYASA), Bengaluru, Karnataka, India; 2 Department of Yoga, Central University of Rajasthan, Ajmer, Rajasthan, India; 3 Head Cataract & Refractive Surgery, Vasan ASG Eye Care Hospital, Kochi, Kerala, India

**Keywords:** dry eye, eye health, meditation, OSDI, QOL, yoga, Trataka

## Abstract

**Background:**

Dry eye significantly impacts visual function, productivity, and quality of life. The personal and economic burden of modern dry eye management is substantial. Yoga, known for its physical and mental health benefits, includes practices such as Jyothi Trataka, a visual concentration exercise that may alleviate dry eye symptoms. Given its minimal resource requirements and ease of implementation, Jyothi Trataka may serve as a cost-effective, accessible adjunct to the management of dry eye.

**Objective:**

This study aimed to evaluate the effectiveness of a 12-week Jyothi Trataka practice in alleviating the signs and symptoms of dry eye.

**Methods:**

A randomized controlled trial with an open-label, parallel-group design was conducted in adults with mild-to-moderate dry eye. A total of 49 participants of both genders, aged 20 to 40 years, with mild-to-moderate dry eye, were randomly assigned to either a yoga group (*n* = 25) or a control group (*n* = 24). Three participants discontinued the intervention, including one from the yoga group and two from the control group, resulting in a final sample of 24 participants in the yoga group and 22 in the control group. All participants provided a medical history and underwent ocular surface and symptom evaluation using standardized protocols at baseline and at the end of the 12-week intervention. The yoga group practiced Jyothi Trataka online for 25 min, 3 days per week, for 12 weeks. The control group received no intervention. Dry eye symptoms were assessed using the Ocular Surface Disease Index (OSDI) score, and the three dry eye signs analyzed were tear breakup time (TBUT), Schirmer’s score, and tear meniscus height (TMH).

**Results:**

Between-group comparisons using the Mann–Whitney U-test revealed significant improvements in the OSDI score (*p* < 0.01) and TBUT (*p* < 0.01) in the yoga group. No significant changes were observed in Schirmer’s score or TMH.

**Conclusion:**

Yoga, specifically Jyothi Trataka, positively affects dry eye symptoms and may be considered a complementary therapy for their management. Longer-duration studies are warranted to explore additional benefits.

**Clinical trial registration:**

https://ctri.nic.in/Clinicaltrials/login.php. Clinical Trial Registry of India (CTRI No. REF/2021/07/045631).

## 1 Introduction

Dry eye is a multifactorial ocular surface disorder characterized by a loss of tear film homeostasis, with associated ocular discomfort and damage. It affects visual function, productivity, and quality of life, and its chronic nature often contributes to psychological distress (1–4).

Globally, the adverse effects of dry eye on productivity have been reported. Recent studies indicate that workers with moderate-to-severe dry eye lose approximately 4–6 h of productive work per week, and workplace performance impairment has been reported in up to 30% of affected individuals (5, 6). Higher symptom severity is associated with marked reductions across physical, psychological, and social quality-of-life (QoL) domains, with QoL scores declining by 20%–30% (7, 8). Collectively, these data indicate that dry eye imposes a substantial functional and socioeconomic burden beyond ocular discomfort alone.

The Tear Film and Ocular Surface Society (TFOS) Dry Eye Workshop (DEWS) II report highlighted the significant socioeconomic burden of dry eye, with rising prevalence linked to increased screen use, air-conditioned environments, and aging populations. Global prevalence estimates range widely from 5% to 50%, with higher rates observed in Asian populations and among women, underscoring its public health significance (9). In North India, the prevalence is reported to be 32%, with individuals aged 21–40 years most affected. Visual display terminal use, smoking, and contact lens use are correlated with a higher risk of developing dry eye (10). The prevalence of dry eye in central India was estimated to be 25% (11).

Current therapeutic approaches for dry eye, including lubricating eye drops, topical anti-inflammatory agents, and punctal plugs, offer symptomatic relief but are frequently constrained by factors such as cost, accessibility, and adverse effects (12). The personal cost of modern dry eye management is substantial and often sustained over long periods, placing a significant financial strain on patients worldwide. Out-of-pocket spending on prescription anti-inflammatory agents, such as cyclosporine or lifitegrast, along with in-office treatments including thermal pulsation or intense pulsed light therapy, can range from several hundred to over a 1,000 U.S. dollars per treatment course, particularly in high-income settings. Even in middle-income regions, these interventions remain costly relative to average income levels (13–15). When recurring expenses for lubricants, supplements, and clinical follow-up are considered, dry eye emerges as a chronic condition with a considerable individual-level economic burden, especially where insurance coverage is limited.

Considering the multifactorial pathophysiology of dry eye, holistic and cost-effective interventions are becoming increasingly pertinent. Yoga, an ancient Indian mind–body practice encompassing cleansing techniques, physical postures, breathing regulation, and meditation, has been associated with benefits for systemic health, including modulation of inflammation, oxidative stress, autonomic function, and psychological wellbeing (16–18). These mechanisms are considered relevant to dry eye, where chronic inflammation, oxidative stress, and autonomic dysregulation play central roles in disease pathophysiology (19).

Among yogic cleansing practices (Shatkarma), Jyothi Trataka is traditionally described as a yogic visual concentration technique performed by steady gazing at a non-flickering flame in a darkened room, followed by palming and relaxation (20, 21). The practice is traditionally thought to strengthen ocular muscles, improve eye health, and promote mental calmness. Empirical evidence supports its potential ocular benefits: a randomized study found that yoga-based eye exercises reduced visual discomfort in computer users (22), while another study reported immediate improvements in cognitive performance following Trataka (23). In addition, yoga ocular exercises, including palming and blinking, have been shown to reduce eye fatigue and improve oculomotor coordination (24). Despite these traditional claims and preliminary findings, rigorous clinical trials evaluating the role of Trataka in ocular disorders, particularly dry eye, remain scarce. Given its simplicity, accessibility, and low cost, Trataka offers a promising adjunctive approach that aligns with integrative and complementary medicine paradigms.

The present randomized controlled trial (RCT) was therefore designed to evaluate the efficacy of Jyothi Trataka in adults with mild-to-moderate dry eye. Specifically, the study assessed changes in subjective symptoms, measured by the Ocular Surface Disease Index (OSDI), and objective clinical outcomes, including tear film breakup time (TBUT), Schirmer’s test, and tear meniscus height (TMH), after a 12-week intervention.

## 2 Materials and methods

### 2.1 Participants and setting

The study was conducted in the Indian state of Kerala between September 2023 and April 2024. In total, 182 participants were screened for eligibility; 60 met the inclusion criteria, of whom 11 declined to participate. A total of 49 participants, of both genders, aged 20 to 40 years, with mild-to-moderate symptoms of dry eye, were randomly assigned to either a yoga group (*n* = 25) or a control group (*n* = 24). Overall, three participants discontinued the intervention, including one from the yoga group and two from the control group. The final sample consisted of 24 participants (16 bilateral dry eye and 8 unilateral dry eye) in the yoga group and 22 participants (20 bilateral dry eye and 2 unilateral dry eye) in the control group.

**Figure 1:**
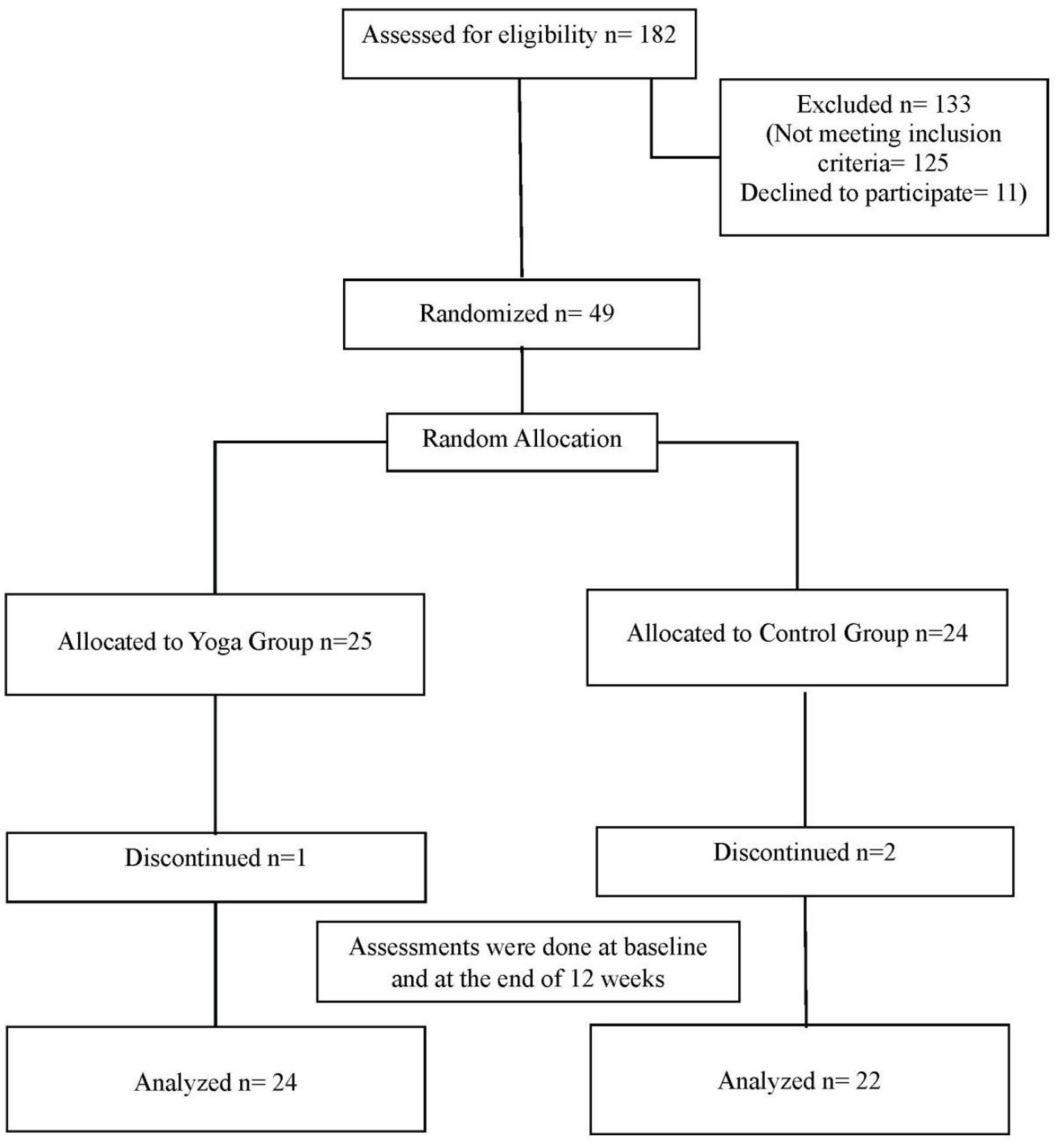
CONSORT flow diagram of the trial.

The yoga group had a female-to-male ratio of 15:9, whereas the control group had 9:13. Their mean ages were 33.00 ± 5.24 years in the yoga group and 30.86 ± 5.50 years in the control group. Baseline characteristics are presented in Table 1. The CONSORT flow diagram of the trial is shown in Figure 1.

**Table 1:** Baseline demographics.

Baseline characteristics	Yoga group			Control group		
Mean age (in years)	Mean ± SD		33.00 ± 5.24	Mean ± SD		30.86 ± 5.50
Age range (in years)	24–39 years			21–39 years		
Sex (count)	Female		15	Female		9
	Male		9	Male		13
Ethnicity	Asian	%	100	Asian	%	100
n (sample size)	24 (16 bilateral dry eye, 8 unilateral dry eye)			22 (20 bilateral dry eye, 2 unilateral dry eye)		
Baseline dry eye signs and symptoms						
Ocular Surface Disease Index (OSDI)	Median (IQR)		17.50 (13.00–22.25)	Median (IQR)		17.00 (15.25–21.50)
Schirmer’s test (mm/5 min)	Median (IQR)		7.00 (5.88–8.13)	Median (IQR)		7.00 (5.63–7.88)
Tear breakup time (s)	Median (IQR)		7.50 (06.38–8.00)	Median (IQR)		7.50 (06.50–07.88)
Tear meniscus height (mm)	Median (IQR)		0.200 (0.15–0.22)	Median (IQR)		0.200 (0.18–0.22)

### 2.2 Inclusion and exclusion criteria

In the initial screening process, participants were asked about their significant medical history. Those who were on continuous medication for any health condition were subsequently excluded from the study. Participants with an OSDI score of ≥13 and ≤33, with the following signs: TBUT score of ≥5 and ≤10s, Schirmer’s score of ≥5 and ≤10 mm, and a TMH of ≤ 0.25 mm, were enrolled (25). In addition, participants were required to use artificial tears at least once daily.

### 2.3 Ethical consideration

Ethical approval was obtained for this study by the Institutional Ethics Committee (IEC) of Swami Vivekananda Anusandhana Samsthana (Approval No: RES/IEC/SVYASA/205/2021). The study procedures were explained, and informed consent was obtained from all participants. The participants were informed that the participation was voluntary, that they could withdraw from the study at any time, and that confidentiality would be maintained.

### 2.4 Study design

A randomized controlled trial with an open-label, parallel-group design was conducted in adults with mild-to-moderate dry eye. The sample size was calculated using G*Power, with an alpha of 0.05 and a power of 0.8. The effect size was 0.52 (26), and the recommended minimum sample size was 15 participants per group. Considering potential dropouts, 25 participants were enrolled in each group and randomized to either the yoga or control group. Randomization was performed using Research Randomizer, an online random sequence generator, by an independent individual not involved in recruitment, intervention delivery, or outcome assessment (27). The yoga group practiced the yogic intervention Jyothi Trataka online for 25 min, 3 days per week for 12 weeks, while the control group did not practice Jyothi Trataka. Outcome measures included dry eye symptoms assessed using the OSDI (28) and three signs of dry eye (Schirmer’s test with anesthesia, TBUT, and TMH) (29, 30). These parameters were measured at baseline and at the end of the 12-week yoga program. The outcome assessor was blinded to group allocation. The trial followed CONSORT guidelines for randomized controlled trials and was registered with the Clinical Trial Registry of India (CTRI) No. REF/2021/07/045631 before participant enrollment.

### 2.5 Assessments

Assessments were conducted using both subjective and objective parameters to comprehensively evaluate dry eye. Subjective assessment included patient-reported symptoms reflecting ocular discomfort and visual disturbance, while objective evaluation comprised clinical signs of dry eye, allowing correlation between symptomatic relief and measurable changes in ocular surface status.

Dry eye symptoms were measured using an OSDI questionnaire. The score ranges from 0 to 100 on the 12-item OSDI, with higher scores indicating greater symptom severity. The minimum clinically important difference (MCID) range for mild-to-moderate was 4.3 to 7.1, and an MCID of 7.1 was used for analysis.

The signs of dry eye were measured separately for each eye (right and left), including Schirmer’s test, TBUT, and TMH. A Schirmer’s test value of 5–9 mm indicates mild-to-moderate dry eye, while < 5 mm indicates severe dry eye. TBUT is measured in seconds, and a value less than 10 s indicates dry eye. A value of 5–9 s is considered mild-to-moderate dry eye, and a value below 5 s is considered severe dry eye; shorter times indicate greater instability of the tear film. The TMH represents the height of the tear layer above the lower lid margin with the eyes open. Lower values strongly suggest quantitative dry eye due to reduced tear production.

### 2.6 Intervention

The 12-week intervention was delivered by an experienced yoga therapist, 3 days per week. All online sessions were conducted under video supervision, with participants’ cameras enabled to allow monitoring of the setting before each session. Participants received standardized pre-session instructions regarding ambient lighting and screen distance, which were verbally reinforced and visually verified during each session. Each Jyothi Trataka session lasted 25 min and consisted of two parts: the first part comprised a set of eye exercises, and the second part involved visual concentration on a non-flickering candle flame positioned at eye level, 2 m from the participants.

The eye exercise session involved eyeball movements in horizontal, vertical, diagonal, and circular directions for 10 min. This was followed by three rounds of visual concentration practice on the candle flame, lasting a total of 15 min. After each round, participants performed palming of the eyes, followed by a quiet sitting period with the eyes closed.

### 2.7 Data analysis

Statistical analyses were performed using JAMOVI software (version 2.4). Data were tested for normality using the Shapiro–Wilk test. As the data were not normally distributed (*p* < 0.05), between-group comparisons were conducted using the Mann–Whitney U-test.

## 3 Results

A total of 182 participants with dry eye were screened; 125 did not meet the inclusion criteria, and 11 opted out of the study. A total of 49 participants were randomly allocated to the yoga group (25 participants) or the control group (24 participants). Three participants dropped out during the intervention, one from the yoga group and two from the control group. The final number of participants was 24 in the yoga group and 22 in the control group. No adverse or harmful events were reported in either group. For participants with bilateral eligible eyes, ocular parameters were averaged across both eyes to obtain a single representative value per participant for statistical analysis.

Data were tested for normality using the Shapiro–Wilk test. As the data were not normally distributed (*p* < 0.05), between-group comparisons were conducted using the Mann–Whitney U-test. There were no baseline differences between the groups for all variables (*p* > 0.05), as assessed using the Mann–Whitney U-test. There was a significant difference between the Jyothi Trataka group and the control group in post-OSDI scores (*p* = 0.001) and TBUT (*p* = 0.002). However, no significant differences were observed between the groups in the post-values of the Schirmer’s score and TMH. The findings from the between-group comparison, conducted using the Mann–Whitney U test, are summarized in Table 2.

**Table 2:** Changes in OSDI score, Schirmer’s score, TBUT, and TMH in the yoga and control groups.

Variables	Yoga group (*n* = 24)		Control group (*n* = 22)		*p*-value		Effect size post (rank biserial correlation)
	Pre	Post	Pre	Post	Pre	Post	
Ocular surface disease index	17.50 (13.00–22.25)	10.00 (6.00–12.50)	17.00 (15.25–21.50)	18.00 (16.00–20.75)	0.472	<0.001	0.807
Schirmer’s score	7.00 (5.88–8.13)	7.00 (6.00–9.13)	7.00 (5.63–7.88)	6.75 (6.13–7.50)	0.956	0.195	0.224
Tear breakup time	7.50 (06.38–8.00)	8.00 (7.00–09.00)	7.50 (06.50–07.88)	7.00 (06.50–07.50)	0.789	0.005	0.479
Tear meniscus height	0.200 (0.15–0.22)	0.200 (0.17–0.23)	0.200 (0.18–0.22)	0.200 (0.15–0.21)	0.825	0.246	0.199

**p < 0.01, Mann–Whitney U-test comparing pre- and post-values of the yoga group with the control group.

Values are presented as median (IQR).

## 4 Discussion

The present study evaluated the effectiveness of Jyothi Trataka, a traditional yogic visual concentration practice, as an adjunct intervention for individuals with dry eye. This practice was chosen for its simplicity, short duration, and feasibility for daily home-based application. The intervention resulted in a significant improvement in subjective symptom scores and a notable increase in tear film stability, as reflected by increased TBUT. In contrast, Schirmer’s scores and TMH did not demonstrate significant changes, suggesting that the observed benefits are primarily related to tear film stabilization and ocular surface functional modulation rather than increased aqueous tear production.

The dissociation between improvements in TBUT and the absence of significant changes in Schirmer’s test is a well-recognized phenomenon in dry eye research, particularly in mild-to-moderate disease (9, 31). TBUT reflects tear film stability, lipid layer quality, blink dynamics, and ocular surface wetting, whereas Schirmer’s test primarily measures basal and reflex aqueous tear secretion (32). Short-term interventions that influence blink efficiency, ocular surface exposure, meibomian gland function, and evaporative loss may, therefore, preferentially improve TBUT without significantly altering aqueous tear production. The findings of this study suggest that Jyothi Trataka may enhance functional aspects of tear film maintenance rather than directly stimulating lacrimal secretion, which may help explain the observed clinical pattern.

One plausible mechanism underlying improvement in TBUT is the modulation of blink dynamics and visual attention. Prolonged screen exposure and modern visual demands are associated with reduced blink frequency and increased incomplete blinking, leading to lipid layer instability and accelerated tear evaporation (33). Mindful visual concentration practices, such as Jyothi Trataka, may promote more complete attach–detach blink cycles, improved ocular surface coverage, and enhanced meibomian lipid distribution, thereby stabilizing the tear film. Supporting this hypothesis, previous studies have demonstrated that blink training and visual attention exercises significantly improve blink patterns, lipid layer behavior, and dry eye symptoms (34, 35). Although blink parameters were not directly measured in the present study, improved blink efficiency offers a clinically plausible explanation for the selective improvement in TBUT.

In addition, ocular microcirculatory modulation may contribute to improved ocular surface homeostasis. Reduced conjunctival and limbal perfusion has been implicated in dry eye pathogenesis by limiting epithelial nutrient delivery and metabolic waste clearance (36). Yogic breathing practices and selected postures have been shown to enhance systemic endothelial function, nitric oxide bioavailability, and peripheral blood flow (37). Enhanced microcirculation could facilitate epithelial repair and meibomian gland function, thereby contributing to tear film stabilization without significantly altering tear volume. However, this hypothesis requires validation through ocular surface–specific perfusion imaging studies.

Beyond these clinically plausible explanations, several biological pathways, including modulation of inflammatory markers, oxidative stress, and autonomic regulation, have been proposed in the broader yoga literature. Dry eye is characterized by elevated inflammatory mediators, oxidative stress markers, and autonomic imbalance (32, 38), and yoga intervention studies suggest a plausible influence on these systemic pathways (17, 18, 39). However, these parameters were not directly measured in the present study, and therefore, any mechanistic interpretation involving these pathways remains speculative. While these mechanisms may plausibly contribute to ocular surface health, definitive conclusions cannot be drawn without targeted biomarker-based investigations in ocular disease–specific populations.

Importantly, autonomic modulation may provide a functional explanation for symptomatic improvement in the absence of increased aqueous tear production. Yoga-based breathing and meditative practices enhance parasympathetic activity and reduce sympathetic dominance (39, 40), potentially improving ocular comfort, blink coordination, tear film distribution, and corneal sensory processing, without necessarily increasing lacrimal gland output. These plausible modulations may explain why subjective symptom relief and TBUT improvement occurred despite stable Schirmer’s and TMH values, a pattern consistent with previous dry eye studies evaluating non-pharmacological interventions.

Taken together, the findings of this study suggest that Jyothi Trataka primarily exerts its benefits through functional modulation of tear film dynamics, blink behavior, and ocular surface comfort, rather than via direct stimulation of tear secretion. While systemic anti-inflammatory and autonomic pathways remain biologically plausible contributors, their roles remain hypothetical and warrant investigation in future mechanistic, ocular surface–specific trials incorporating biomarker analysis, blink metrics, and ocular perfusion imaging.

## 5 Strengths and limitations

The primary strength of this study lies in its randomized controlled design and its potential to offer a low-cost, accessible intervention for dry eye symptom relief. Potential sources of bias include selection bias arising from recruitment within a specific population and voluntary participation, which could limit the generalizability of the findings. The open-label design, with no blinding of participants and data analysts, represents an important methodological limitation and may have contributed to expectancy-related and detection biases, particularly affecting subjective outcomes. Future trials using single-blind or assessor-blinded designs are warranted to strengthen the validity of these findings.

Furthermore, the relatively short intervention duration and absence of periodic follow-up assessments may have limited the evaluation of sustained or delayed effects. Future studies should investigate the effects of longer practice durations and incorporate periodic evaluations for a more comprehensive understanding of objective outcomes. The use of a no-intervention control group represents another important limitation of this study, as it does not fully account for non-specific effects such as time, attention, expectancy, and environmental influences, which could have contributed to the observed outcomes, particularly for subjective measures. Future studies incorporating active or sham comparator interventions are warranted to better isolate the specific therapeutic effects of the intervention.

## 6 Future directions

Future studies should focus on larger, randomized trials with longer follow-up periods to confirm the durability of the observed benefits of Jyothi Trataka in dry eye. The inclusion of objective biomarkers and patient-reported outcomes may help clarify the underlying mechanisms and clinical relevance. Comparative studies evaluating Jyothi Trataka as an adjunct to standard therapies could further define its role in the comprehensive management of dry eye.

## 7 Conclusion

The findings of this randomized controlled trial suggest that Jyothi Trataka is associated with improvements in patient-reported symptoms and tear film stability in mild-to-moderate dry eye. As a simple practice requiring minimal infrastructure and no specialized equipment, Trataka may offer a simple and economically sustainable adjunct to conventional dry eye care. While benefits were observed primarily in subjective outcomes and TBUT, the lack of change in aqueous tear parameters underscores the need for further investigation. Larger trials with longer follow-up periods are needed to confirm these findings and to better define the role of yogic visual concentration practices in comprehensive dry eye management.

## Data Availability

The raw data supporting the conclusions of this article will be made available by the authors, without undue reservation.
